# MonkeyCBP: A Toolbox for Connectivity-Based Parcellation of Monkey Brain

**DOI:** 10.3389/fninf.2020.00014

**Published:** 2020-04-28

**Authors:** Bin He, Zhengyi Yang, Lingzhong Fan, Bin Gao, Hai Li, Chuyang Ye, Bo You, Tianzi Jiang

**Affiliations:** ^1^School of Mechanical and Power Engineering, Harbin University of Science and Technology, Harbin, China; ^2^Brainnetome Center, Institute of Automation, Chinese Academy of Sciences, Beijing, China; ^3^National Laboratory of Pattern Recognition, Institute of Automation, Chinese Academy of Sciences, Beijing, China; ^4^University of Chinese Academy of Sciences, Beijing, China; ^5^Key Laboratory for NeuroInformation of the Ministry of Education, School of Life Sciences and Technology, University of Electronic Science and Technology of China, Chengdu, China; ^6^CAS Center for Excellence in Brain Science and Intelligence Technology, Institute of Automation, Chinese Academy of Sciences, Beijing, China; ^7^Queensland Brain Institute, The University of Queensland, Brisbane, QLD, Australia; ^8^Chinese Institute for Brain Research, Beijing, China

**Keywords:** parcellation, brain atlas, neuroimaging pipeline, diffusion tractography, parallel computing

## Abstract

Non-human primate models are widely used in studying the brain mechanism underlying brain development, cognitive functions, and psychiatric disorders. Neuroimaging techniques, such as magnetic resonance imaging, play an important role in the examinations of brain structure and functions. As an indispensable tool for brain imaging data analysis, brain atlases have been extensively investigated, and a variety of versions constructed. These atlases diverge in the criteria based on which they are plotted. The criteria range from cytoarchitectonic features, neurotransmitter receptor distributions, myelination fingerprints, and transcriptomic patterns to structural and functional connectomic profiles. Among them, brainnetome atlas is tightly related to brain connectome information and built by parcellating the brain on the basis of the anatomical connectivity profiles derived from structural neuroimaging data. The pipeline for building the brainnetome atlas has been published as a toolbox named ATPP (A Pipeline for Automatic Tractography-Based Brain Parcellation). In this paper, we present a variation of ATPP, which is dedicated to monkey brain parcellation, to address the significant differences in the process between the two species. The new toolbox, MonkeyCBP, has major alterations in three aspects: brain extraction, image registration, and validity indices. By parcellating two different brain regions (posterior cingulate cortex) and (frontal pole) of the rhesus monkey, we demonstrate the efficacy of these alterations. The toolbox has been made public (https://github.com/bheAI/MonkeyCBP_CLI, https://github.com/bheAI/MonkeyCBP_GUI). It is expected that the toolbox can benefit the non-human primate neuroimaging community with high-throughput computation and low labor involvement.

## Introduction

Non-human primates (NHPs) resemble high similarities in the neuroanatomical structures and cognitive functions to humans (Perretta, 2009). NHP models are essential in understanding brain structures and functions, as well as neurodegenerative diseases and pathological disorders. The neuroimaging on NHPs has provided vital information in basic and translational neuroscience research for various diseases, such as Parkinson’s disease (Zhang et al., 2000), Alzheimer’s disease (Smith et al., 1999), and autism (Amaral et al., 2003). There is an increasing interest in magnetic resonance imaging (MRI) of monkeys for neuroscience research, imposing a significant challenge in high-quality brain atlas constructed from MRI data.

Building an atlas involves dividing the brain into regions with certain homogeneity within each region. The definition of region boundaries has always been challenging since the development of the well-known Brodmann atlas (Brodmann, 1909). Various atlases have been constructed from MRI (Toga et al., 2006; Amunts and Zilles, 2015; Fan et al., 2016; Glasser et al., 2016), and they diverge in the boundary defining criteria, ranging from cytoarchitectonic features, neurotransmitter receptor distributions, myelination fingerprints, transcriptomic patterns to structural and functional connectomic profiles, and any combination of the above. Among them, connectivity-based parcellation (CBP) has attracted increasing interest from the community. The rationale behind CBP is that the function of a certain cortical area is mainly determined by the unique connectional pattern defined by inputs and outputs (“connectional fingerprint”), and its local infrastructure characterized by microstructural properties (Passingham et al., 2002). Therefore, brain areas should be definable by aggregating voxels/vertices demonstrating similar connectivity patterns, characterized by structural, functional, or meta-analytic connectivity (Behrens et al., 2003; Kim et al., 2010; Eickhoff et al., 2011), and so forth into clusters. Researchers have used CBP to form cartographic maps of specific brain regions or the entire cortex (Eickhoff et al., 2015), and the whole human brain—the human brainnetome atlas (Fan et al., 2016)—which is based on the anatomical connectivity profiles derived from structural neuroimaging data.

A brain atlas of macaque monkey has been constructed using a CBP pipeline similar to Automatic Tractography-Based Brain Parcellation (ATPP) (Wang et al., 2018). However, previous work involves many operator interventions throughout the process, including manual delineation of brain tissue, fine-tuning registration, and fiber tracking parameters. To facilitate researchers with limited programming skills in using CBP, we have published an automatic pipeline for building brainnetome atlas of human brain, named ATPP. In this paper, we present a variation of ATPP, which is dedicated to monkey brain parcellation, to address the significant differences in the process between the two species. The new toolbox, MonkeyCBP, has major alterations in three aspects: brain extraction, image registration, and validity indices. By parcellating two different brain regions [posterior cingulate cortex (PCG) and frontal pole (FP)] of the rhesus monkey, we demonstrate the efficacy of these alterations.

## Framework of MonkeyCBP

### Overview

**FIGURE 1:**
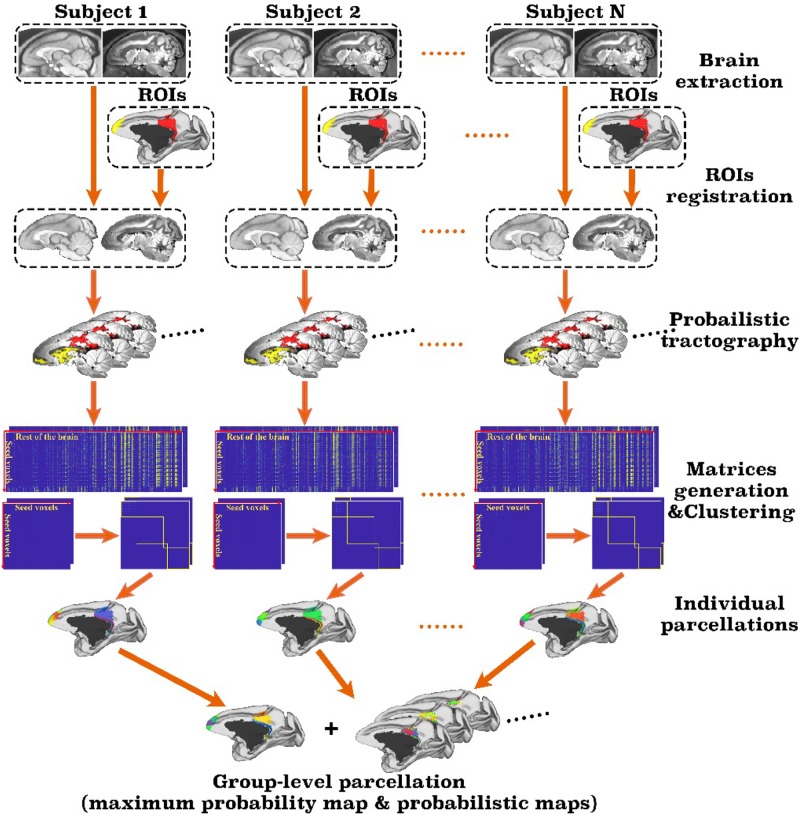
Framework of the MonkeyCBP toolbox. The processing steps of the pipeline mainly including brain extraction, registration, probabilistic tractography, and clustering. First, the technique of brain extraction for monkey MRI data was performed; then based on T1w and diffusion tensor images of the same subjects, two defined regions of interest (ROIs), including the right posterior cingulate cortex and the bilateral frontal pole, are parcellated simultaneously. After pipeline processing, the parcellation results at both individual level and group level with a maximum probability map and probabilistic maps of each subarea of the right posterior cingulate cortex and the bilateral frontal pole are generated.

The toolbox takes single or multiple user-defined regions of interest (ROIs) and a set of parameters as input and automatically parcellates the brain and output the parcellation results, as well as debug information in a text file. Figure 1 is the flowchart of the toolbox. As a pipeline tailored for monkey brain, MonkeyCBP toolbox has several steps that are significantly different from the ATPP package. First, two methods for monkey brain extraction have been incorporated to the toolbox. One is a modified version of the Brain Extraction Tool (BET) method (Luo et al., 2018); the other is based on multi-atlas segmentation (MaS). Second, the SPM registration method used for human brain has been replaced by methods based on Advanced Normalization Tools (ANTs)^1^ (Avants et al., 2011), which are more suitable for monkey brain processing. Third, the parcellation validity indices have been improved. In addition to preserving the previous verification method on the basis of the information of standard space, we introduced a statistical framework method of principal component analysis (PCA), which is simple and effective to facilitate users to select cluster numbers and further improves the reliability of the conclusion by analyzing the original data, to verify the results of brain parcellation.

### Brain Extraction

Brain extraction, or skull stripping, from human MRI data has been widely examined in the development of neuroimaging, as a stepping stone for subsequent analyses, such as intracranial volume calculation, tissue classification, subregion segmentation, connectivity computation, and brain network construction. Nevertheless, the technique of brain extraction for monkey MRI data has not been as mature as that for human data. The task of brain extraction is to retain the brain tissue only and remove the non-brain tissues, including scalp, skull, and eyeballs, which often negatively affect automated image registration, segmentation, and further analyses. As reviewed in Kalavathi and Prasath (2016), human brain extraction methods can be broadly classified into four categories on the basis of morphology, intensity, deformable surface, and atlas/template. In addition, hybrid methods combining any of the four types have also been explored.

We have reported a BET (Smith, 2002) variation for rhesus brain extraction (Luo et al., 2018). Hereby, we briefly review a subset of the atlas/template-based and hybrid methods, in which multiple atlases are used. An atlas is defined by a pair of structural images and its manual segmentation, or labels. Besides preprocessing (e.g., intensity inhomogeneity correction) and postprocessing (e.g., morphological hole filling), MaS involves three major steps:

• label mapping: registering the images in multiple atlases to the image under segmentation and transforming the manual labels accordingly;
• atlas selection: choosing the atlases best matched to the image under segmentation; and
• label fusion: fusing the multiple transformed labels to obtain the final segmentation.

General-purpose MaS methods were reviewed in Iglesias and Sabuncu (2015) and was initially introduced to neuroimaging for segmenting brain into anatomical structures (Aljabar et al., 2009), and it was demonstrated that image similarity and age were both suitable for atlas selection. Artaechevarria et al. (2009) prompted local weighting scheme for label fusion and highlighted that not a single fusion method was optimal to all structures. Lötjönen and colleagues reported a hybrid method combining MaS and expectation maximization (Lötjönen et al., 2010). Wang J. et al. (2014) used a graph-based atlas selection method to reduce overall computational time. A recent advance was the sparsity-based atlas selection strategy applied to newborn brains (Serag et al., 2016).

Dedicated to brain extraction, Leung et al. presented a multi-atlas propagation and segmentation (MAPS) method (Leung et al., 2011) that outperformed three methods without using atlases, including BET. BEaST was proposed to speed up the time-consuming label mapping step by reducing the areas to be applied patch-based segmentation, the number of atlases, and patches (Eskildsen et al., 2012). Doshi et al. (2013) developed a method called Multi-Atlas Skull Stripping (MASS), to enhance atlas representativeness by defining study-specific atlases selected from the images under segmentation, rather than existing atlases. In their method, extra effort is needed for semi-automatically segmenting the selected images. Joint label fusion (JLF) was proposed to overcome the invalid assumption that the atlases are independent, and significant improvement was reported (Wang et al., 2013). MaS methods were also adapted to segment fetal brains (Tourbier et al., 2015) and brain/ventricles simultaneously (Tang et al., 2015).

A variant of BET was developed for rhesus monkey brain extraction and achieved a dice similarity coefficient (DSC) of 92.6% (Fu et al., 2016). However, there has been limited work on the use of MaS approaches for rhesus monkey brain extraction. Ballanger et al. applied MaS method to extract brain from T1-weighted (T1w) MR images of crab-eating monkeys (*Macaca fascicularis*) (Ballanger et al., 2013), and they reported the first MaS dataset for NHP. Maldjian et al. (2016) presented study-specific atlases for rhesus, vervet, and cynomolgus monkeys. Brain extraction was used to transform the labels in the best performed atlas judged by image similarity; no quantitative evaluation was reported though. A hybrid method combining MaS for coarse extraction with surface deforming guided by local intensity and priors was developed and tested on both human and NHP brain images (Wang Y. et al., 2014). Tested on a single dataset of 20 adult healthy rhesus macaques, it achieved overall DSC of around 97%, mean absolute surface distance (MASD) of around 0.6 mm, and maximum surface distance of around 4 mm, which significantly outperformed Brain Surface Extractor (BSE), Hybrid Watershed Algorithm (HWA), Analysis of Functional NeuroImages (AFNI), and BET and its two variations.

In this study, we developed a MaS-based protocol for automatically extracting brain tissues from structural MRI data of rhesus macaque (*Macaca mulatta*). We evaluated the performance by comparing the results against manual segmentations. The brain tissue boundaries of all subjects were manually traced on the axial view using ITK-SNAP (Yushkevich et al., 2006), whereas the other two views were displayed for reference. Manual delineations along with original images were visually inspected by a co-author slice by slice to rectify incorrectly segmented regions, recover over-segmented brain tissues, and remove non-brain tissues. Thus, an atlas was obtained for each subject from its T1w MR image, with brain tissue voxels manually labeled as 1 and others as 0. Then the three major steps of MaS were followed: (i) For label mapping, the software package ANTs was used for non-linear registration because of its outstanding performance (Klein et al., 2009). (ii) For atlas selection, we built study-specific atlases for D24 and D30, using a leave-one-out (LOO) cross-validation (CV) scheme. In each LOO CV run, for each subject, the manual segmentations of the remaining subjects were non-linearly registered and mapped to its space. The label fusion methods were then applied to combine the mapped segmentations and extract the brain of the left-out subject. (iii) For label fusion, we tested majority voting (MV) (Lötjönen et al., 2010) and JLF (Wang et al., 2013). To shorten the overall time, we applied label fusion to voxels within a mask around the brain boundary, which was generated by subtracting the intersection of all mapped atlas segmentations from their union.

**FIGURE 2:**
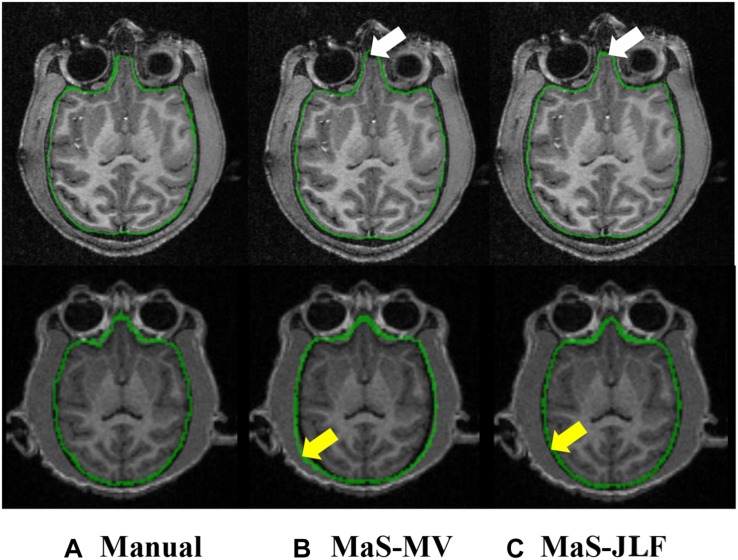
Example T1 images from D24 **(top)** and D30 **(bottom)**, overlaid with manual segmentation, MaS-MV, and MaS-JLF from left to right. Arrows indicate differences in segmentations. MaS, multi-atlas segmentation; MV, majority voting; JLF, joint label fusion.

**FIGURE 3:**
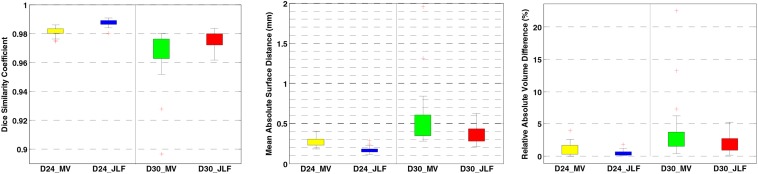
Brain extraction evaluation results on D24 and D30. From left to right: DSC, MASD, and RVD. DSC, dice similarity coefficient; MASD, mean absolute surface distance; RVD, relative volume difference; MV, majority voting; JLF, joint label fusion.

Two independent datasets (24 and 30 subjects, named D24 and D30, respectively) were used for testing the generalizability of the developed pipeline. The accuracy was evaluated using DSC, MASD, and relative volume difference (RVD) measures (Yang et al., 2015) with manual segmentations as reference. We visually inspected the segmentation results of both MaS methods, and we found that all images (54 in total) were reasonably segmented without obvious failure, indicated by DSC values (108 in total) larger than 0.85. As illustrated in Figures 2, 3, JLF significantly outperformed MV on both datasets, in terms of DSC, MASD, and RVD (paired *t*-test, *p* < 0.05). The quantitative evaluation is shown in Table 1. The extractions on D24 are generally better than D30. However, the maximum symmetric surface-to-surface distance of D24 (2.97 ± 2.83 mm) is notably larger than that of D30 (1.94 ± 1.90 mm), designating worse extreme case of local segmentation error in D24 than D30.

**TABLE 1:** Quantitative evaluation of the tested methods on the two datasets.

**Data**	**Method**	**DSC**	**Specificity**	**Sensitivity**	**MASD (mm)**	**RVD (%)**
D24	MaS-MV	0.981 ± 0.003	0.981 ± 0.007	0.999 ± 0.001	0.27 ± 0.057	1.14 ± 1.01
	MaS-JLF	0.987 ± 0.002	0.987 ± 0.004	0.999 ± 0.000	0.17 ± 0.042	0.50 ± 0.42
D30	MaS-MV	0.967 ± 0.017	0.970 ± 0.019	0.996 ± 0.005	0.54 ± 0.34	3.90 ± 4.32
	MaS-JLF	0.976 ± 0.006	0.976 ± 0.013	0.997 ± 0.013	0.36 ± 0.11	1.92 ± 1.34

*DSC, dice similarity coefficient; MASD, mean absolute surface distance; RVD, relative volume difference.*

### Registration

Registration is a critical step in CBP for generating probability maps of parcellations. For each subject in a cohort, the skull-stripped structural and diffusion-weighted imaging (DWI) data are transformed to a standard template (e.g., INIA19 structure template) using linear and non-linear registration algorithm, to obtain the forward and inverse transformations. CBP starts with transforming a predefined ROI in the standard space, either extracted from an existing atlas or drawn manually, into a seed mask in the individual DWI space using the inverse transformation. After the seed mask is parcellated, the forward transformation is employed to transform the parcellation from the individual space to the standard template space. In MonkeyCBP toolbox, the image registration between the individual and the template space is performed using ANTs (see text footnote 1), owing to its superior performance (Nazib et al., 2018) in deformable registration evaluated on large-scale datasets (Avants et al., 2011). There are many strategies, ranging from linear algorithms to diffeomorphic algorithms, in ANTs that use different transformation models and similarity metrics and their combinations to improve the performance and alleviate over-fitting problems in various application scenarios. For example, symmetric diffeomorphic image registration of ANTs was proven to be effective for MRI registration with large deformation (Avants et al., 2008) and algorithm parameters, such as regularization terms, could be fine-tuned to prevent over-fitting. In addition, to quantitatively evaluate the registrations results, three different similarity measurements (mean square difference, cross-correlation, and mutual information) were used in MonkeyCBP. The similarity metrics between the registered image pairs were calculated, and if they were below a threshold determined on the basis of experience, the toolbox would issue a warning and request human inspection.

### Probabilistic Tractography

Probabilistic diffusion tractography is implemented in FSL by taking repetitively samples many times from the distribution of voxel-wise streamlines to generate the connectivity probability. All streamlines are synchronously yield to the orientations sampled from the diffusion distributions produced by bedpostx tool, as expounded in Behrens et al. (2007). Subsequently, the connectivity profiles at whole-brain level for each voxel in the seed mask are generated in the form of image. For each single image, it represents a set of connectivity value between that voxel and all brain voxels, and per-connectivity value of the voxel represents the number of true streamlines that pass through that voxel. The connection probability between voxels is defined as the connectivity value divided by the total number of streamlines sampled from the seed voxel. In order to compensate for the deviation caused by distance from the seed mask, the connectivity distribution is corrected using the length of the pathways (Tomassini et al., 2007; Mars et al., 2011). A curvature threshold is used to exclude implausible pathways by limiting the angle between two steps (Thomas et al., 2014). Finally, a small threshold value is employed to discard the sample values with a small number of streamlines (Makuuchi et al., 2009; Sinke et al., 2018). By applying this fixed threshold, it enables the images not only to have fewer spurious connections but also to retain enough sensitivity to the true connections (Heiervang et al., 2006; Johansen-Berg et al., 2007). For monkey data, compared with the parameter settings from previous process from ATPP in this step (Li et al., 2017), MonkeyCBP re-recommended new parameter values (e.g., number of samples - default = 10,000; Steplength in mm - default = 0.2) (Thomas et al., 2014; Folloni et al., 2019; Sani et al., 2019). To facilitate individual brain parcellation, the down-sampling rate of the connectivity profile in subsequent steps is also suggested to be modified (e.g., 3-mm isotropic voxels) (Johansen-Berg et al., 2004).

### Individual Parcellation, Probabilistic Maps, and Maximum Probability Maps

Based on the native connectivity matrix, a cross-correlation matrix was calculated and fed into a spectral clustering algorithm for the parcellation of each individual brain region. The maximum number of clusters *K* was decided by the experimenter, and the clustering results can be generated as a range from 2 to *K* in MonkeyCBP at a draught, which is flexible to facilitate making decisions for final parcellation. Then the corresponding clusters of individual subjects are transformed into the template space using previous transformation profiles. Owing to the cluster label of clustering algorithms for each subject is random, we attempt to find a solution to make the parcellation results above comply with the most consistent labeling scheme. First, the labeling schemes of each individual are all incorporated into a thresholded group-level cross-correlation matrix. Each element in the matrix represents the connection similarity between any two voxels in ROI. Second, the spectral clustering algorithm is employed again for the similarity matrix to generated a group-level labeling result. Finally, the labeling result is propagated back to all the individual cluster using an assignment algorithm that ensures maximum spatial overlap (Munkres, 1957). Furthermore, in virtue of convergent evidences from previous studies (Brodmann, 1909; Petrides and Pandya, 2002; Chen et al., 2012; Bludau et al., 2014; Cui et al., 2016), it also supports the topological homology of bilateral brain. Scilicet, if the ROIs are the corresponding regions of bilateral hemispheres, the label consistency across hemispheres is guaranteed before the labeling scheme is propagated. Considering interindividual variability in the parcellation results of the ROI, MonkeyCBP calculated the maximum probability map (MPM), which quantitatively reflects which cluster a given voxel of the ROI most likely belonged, of each subregion across all the subjects to indicate the final results (Eickhoff et al., 2005, 2006; Caspers et al., 2008). Subsequently, a smoothing step (Wang et al., 2012) is applied to correct the noisy voxels whose labels are different from most labels of the six immediate neighbors.

### Validity Indices

Determining the optimal number of clusters is always a fundamental and challenging issue in partitioning clustering. The optimizing for number of clusters is somehow subjective, and there is no precise answer. To refrain from arbitrarily choosing the number of the subdivisions, previous ATPP has provided many effective verification indicators on the basis of the data in standard space. Based on ATPP, MonkeyCBP not only retained the verification indices but also added the analysis of the original connectivity data in diffusion space. In MonkeyCBP, we introduce the PCA-based statistical models for determining *k* of the optimal solution, which further constrains the choice of clustering numbers and effectively complements the shortcomings of previous validation methods.

#### Consistency Criterion

The consistency criteria mainly consist of three aspects: (1) consistency across parcellations criterion, (2) consistency within parcellation criterion, and (3) consistency of topology criterion, which are consistent with those of a previous study (Li et al., 2017). In brief, the first criterion contains Dice coefficient (Dice, 1945), Cramer’s *V* (Cramér, 1946), normalized mutual information (Witten et al., 2017), and variation of information (Meila, 2003); the second criterion contains averaged silhouette value (Rousseeuw, 1987) and continuity index (Li et al., 2017; Wang et al., 2018); the third criterion contains consistency of topology criterion, hierarchical index (Kahnt et al., 2012), and topological distance index (Tungaraza et al., 2015); and the detailed descriptions are in keeping with ATPP.

#### Principal Component Analysis

PCA, as one of the simplest and robust ways to analyze multidimensional data, is a powerful statistical framework for the analysis of tractography-based parcellation (Thiebaut de Schotten et al., 2014; Cerliani et al., 2017) and has become increasingly popular recently in different studies (Markiewicz et al., 2011; Craddock et al., 2012; Leonardi et al., 2013; Tian et al., 2013; Nayal et al., 2014; Smith et al., 2018). Based on the theory of DWI and tractography techniques, the whole-brain connectivity of each voxel is deemed as multivariate dataset in the process of individual brain parcellation. Generally, this method allows identifying *k* components on the basis of *k* initial attributes and further determines the number of the subregions without *a priori*. Referring to a large number of the previous studies (Kaiser, 1960; Cattell, 1966; Wold, 1978; Jolliffe, 1986; Ferré, 1995; Teipel et al., 2007; Brown, 2009; Thiebaut de Schotten et al., 2014; Ledoit and Wolf, 2015; Rea and Rea, 2016; Pasini, 2017), MonkeyCBP offers three criteria for estimating the number of clusters to select for each subject: (1) cumulative contribution, (2) eigenvalues, and (3) scree test. Statistical analysis was performed using MATLAB, and all of the connectivity matrices for each animal derived from the data of the probabilistic tractography were fed into PCA to calculate the above indicators. First, the cumulative contribution, which means cumulative proportion of the variation explained, is an indispensable benchmark to determine the principal components for its simplicity and effectiveness. As usual sort of standards, the threshold often varies between 70 and 90%, which depends on the context. The high threshold means loose selection of clustering number subsequently, which means more choice for the number of clusters. However, by investigating a large number of studies, the minimum threshold that was suggested should be greater than 70%. Second, only factors with eigenvalues greater than 1 are retained. Finally, under criteria 1 and 2, a scree test was performed for each subject with their eigenvalues to separate the principal from residual components (Cattell, 1966). A power curve was plotted by fitting the data derived from the probabilistic tractography, and the inflexion point was extracted for each subject as the number of principal components using a homemade routine written in MATLAB R2017. Then all the subjects were averaged to obtain a mean value, and a fixed clustering number or very limited integer values within a narrow range were estimated as a guide to group together ROIs for the bilateral hemispheres.

#### Determination of the Optimal *K* Solution

A key step in the MonkeyCBP pipeline is to identify the optimal number of clusters *k*. Data clustering have been intensively developing for last decades; however, it still is a long-standing ill-posed problem intrinsically where the goal is to partition the data into some unknown number of clusters on the basis of inherent information alone (Jain, 2010). It is very difficult to select an intrinsic number of clusters because of the data-driven nature of clustering, and there is no definitive answer to this question. In general, these methods for determining the optimal clustering number include direct methods that consist of optimizing a criterion, statistical testing methods that consist of comparing evidence against null hypothesis, and decisions based on investigators, that is, cluster validity criteria. ATPP provides a variety of effective indicators to facilitate user selection. There is no doubt that these validity indices are very effective; in spite of this, previous methods primarily focused on the analysis of data in standard common space. It means that the input data are mainly the results of the registration of individual results into the standard space, whereas information contained in the data of individual diffusion space was not fully utilized, especially the most fundamental connectivity profiles. Although the method is proved to be effective in building the human brainnetome atlas, it requires high quality of original data itself and high sensitivity to the accuracy of registration process, transforming individual image to the common space. In this context, MonkeyCBP provides another statistical testing method based on PCA, exploiting the information in individual space. To a large extent, PCA could help users directly remove the unnecessary extremal points and in turn verify the previous indicator results offered by ATPP. Finally, on the basis of all the above indicators, users will be recommended a more detailed optimization scheme.

## Implementation

### Overview

**FIGURE 4:**
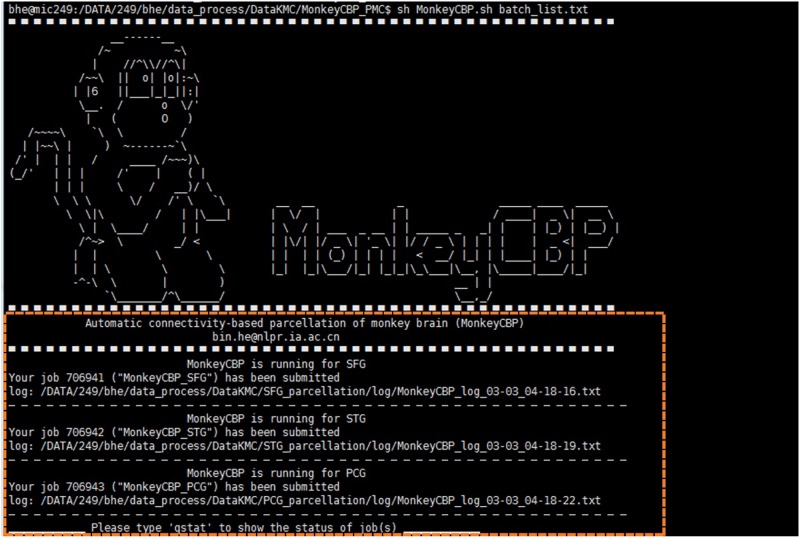
Command line (CLI) version of MonkeyCBP. CLI version is multi-region of interest (ROI) oriented; therefore, users could parcellate multiple brain regions synchronously. The diagram shows that the user submitted three concurrent tasks for the parcellations of superior frontal gyrus (SFG), superior temporal gyrus (STG), and posterior cingulate gyrus (PCG) at the same time.

**FIGURE 5:**
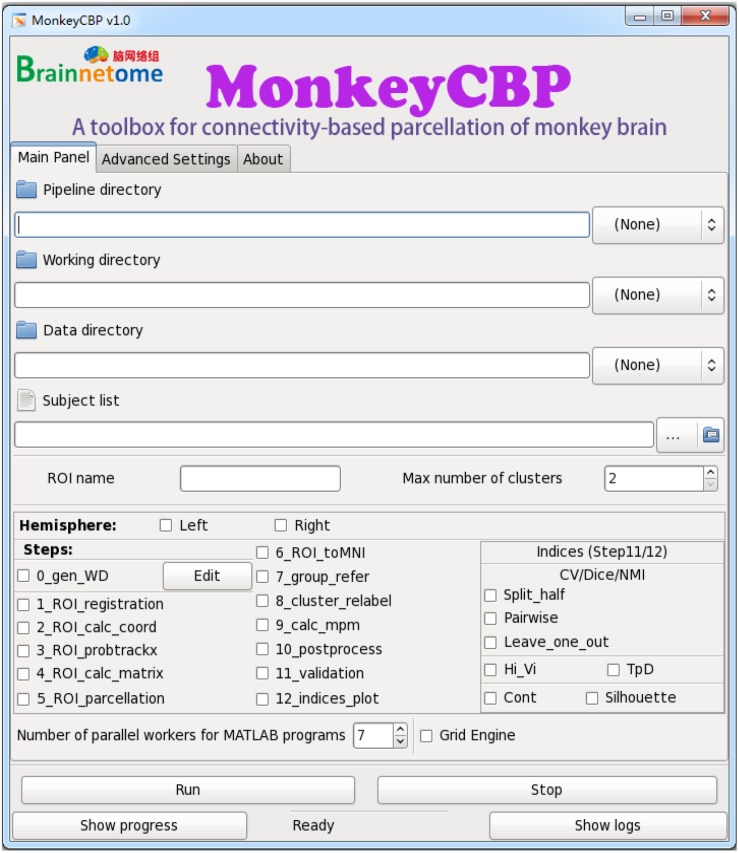
Graphical user interface (GUI) version of MonkeyCBP. The “Main Panel” menu including settings for input files and directories as well as configuration information. The “Advanced Settings” menu including advanced parameters for the command path and file as well as some specific parameters. The “About” menu displays the information relevant to the developer and license. At the bottom of the interface are several fixed buttons designed for users to control the start-up and shutdown of tasks, as well as to check the running progress and processing logs in real time. In addition, MonkeyCBP GUI version also supports parallel computing, but it is just single-ROI oriented, which is not like the CLI version that could parcellate multiple brain regions at the same time.

Compared with ATPP, MonkeyCBP is mainly devoted to the parcellation of monkey brain and introduces the process of brain extraction. More specifically, the tractography-based monkey brain parcellation pipeline also consists of 12 steps, in which the methods of image registration and the final verification parts are modified respectively. Meanwhile, the brain extraction is added before the brain parcellation pipeline in MonkeyCBP. In addition, the users with different programming skills could choose the command line (CLI) version (Figure 4) and graphical user interface (GUI) (Figure 5) version to use according to their preferences.

From the implementation perspective, the tractography-based parcellation of monkey is mainly split into the following steps:

(1) Preprossing: Brain extraction, eddy current correction, DTIFIT, Bedpostx.
(2) ROI registration: The registration is performed between the data of individual space and standard space, and then ROI of standard space is registered to individual diffusion space.
(3) Probabilistic tractography: For the registered ROI of each subject in diffusion space, a plain text that contains the *xyz* coordinates of all the voxels in the seed mask is obtained; subsequently, probabilistic tractography is performed.
(4) Individual parcellation: For each registered ROI, a cross-correlation matrix is calculated based on the results of probabilistic tractography, and then clustering algorithm is applied to parcellate the registered ROI.
(5) Group results: The parcellation results are inversely transformed from individual diffusion space to the standard template space. For each subdivision, probabilistic maps and the maximum probability map across subjects are generated.
(6) Validity indices: Various validity indices of the parcellation are computed and the graphs that depict the trends of these indices are generated.

### Prerequisites

Before running MonkeyCBP, users must check the following prerequisites. (1) Input data. First, make sure the images are in correct orientation. If not, we recommend a freely available Medical Image Processing, Analysis, and Visualization (MIPAV)^2^ (McAuliffe et al., 2001) toolbox to reorient the image. Second, MonkeyCBP requires non-DWI (b0) and those images preprocessed by bedpost (part of the FSL software package) for all the subjects. (2) Environment and tools. Comparing the requirements of environment and software tools, MonkeyCBP is mostly similar to ATPP. Note that SPM has been replaced with ANTs for the image registration. Other major computational environment configurations are the same as ATPP, for both GUI and CLI versions of MonkeyCBP.

### Directory Structure and File Naming Conventions

A simple, normative, and consistent directory structure enables pipeline software more flexible to plan, schedule, and check jobs, which will dramatically improve its work efficiency. MonkeyCBP uniformly creates the initialized working directory for the ROI of each subject that contains the following: (1) a template subdirectory including the files for registration and the predefined ROIs, (2) a log subdirectory covering the running logs of each step, and (3) subject_id subdirectories comprising the images of each subject (e.g., T1w/T2w image, b0 image, or FA image). After the software starts, a string of intermediate results and logs with specific and uniform names will be produced.

### Modular and Hierarchical Structure of the Implementation

In order to improve the software easily and flexibly, the core algorithms of per process in MonkeyCBP are modular in design. In MonkeyCBP, the top-level script of CLI version, or callback functions of GUI version, is responsible for reading the configuration file and submitting jobs. The second-level script is applied to activate a series of predefined procedures and record running logs. The third-level scripts are used to perform specific jobs, employing either internal MATLAB functions or third-party programs.

### Implementation Details

MonkeyCBP actualizes parallel computing within or across machines *via* SGE and MATLAB PCT, which ensured the efficiency of the software. In addition, users can freely choose between two software versions. First, MonkeyCBP CLI version comprises a standard set of hierarchical bash shell scripts that glue together the codes of MATLAB and/or third-party programs. A list file of TXT format defines a series of inherent information (data directory, list of subjects, working directory, ROI name, and maximum number of subregions) of one brain region in per row. The top script, which named MonkeyCBP.sh, submits jobs that each consists of a second-level script, pipeline.sh, and the information about ROI as well as the configuration parameter in config.sh, to opportune machines across the cluster. The third-level scripts are triggered to perform specific works by the second-level scripts that produce the processing logs for users to debug and examine the results. If the user’s hardware supports graphics processing unit (GPU) computing, we also provide an interface of GPU to speed up the data processing process. Second, the other version of MonkeyCBP with a GUI is convenient for users with few programming skills. Based on GTK-server, it is designed with three menus: (1) “Main Panel,” (2) “Advanced Settings,” and (3) “About.” The “Main Panel” menu includes settings for input files and directories as well as configuration information. The “Advanced Settings” menu includes advanced parameters for the command path and file as well as some specific parameters. The “About” menu displays the information relevant to the developer and license. At the bottom of the interface are several fixed buttons designed for users to control the start-up and shutdown of tasks, as well as check the running progress and processing logs in real time. In addition, MonkeyCBP GUI version also supports parallel computing, but it is just single-ROI oriented, which is not like the CLI version that could parcellate multiple brain regions at the same time.

## Results and Discussion

In the current study, we developed a toolbox named MonkeyCBP for CBP of monkey brain with automatic processing and large-scale parallel computing. MonkeyCBP provides two skull-stripping methods dedicated for rhesus monkey. The CLI version implements simultaneous parcellation of multiple brain regions, and the user-friendly interactive GUI version offers the parcellation for a single brain region.

**FIGURE 6:**
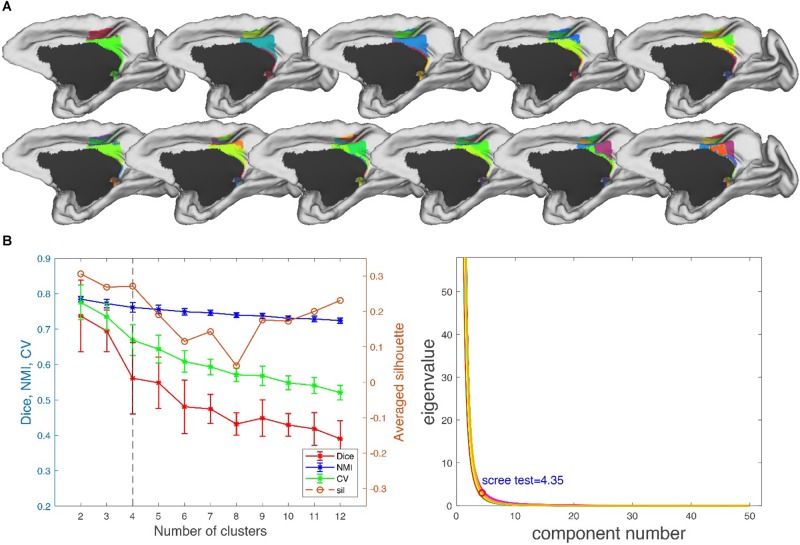
Parcellation results of right posterior cingulate gyrus (PCG.R). **(A)** The maximum probability maps (MPMs) of PCG. R with 2–12 clusters. Note that there is no consistency among subareas with the same color in different results. **(B)** Validity indices of the parcellation results for PCG. R using leave-one-out resampling technique with 200 repetitions (left) and principal component analysis (PCA) (right). Thereinto, the relative higher value of Dice, NMI, CV, and sil hint the more consistent result across the whole parcellations. The error bars represent standard deviation. The optimal result of 4 clusters seems the most reasonable according to these indices, which is consistent with the result of PCA.

**FIGURE 7:**
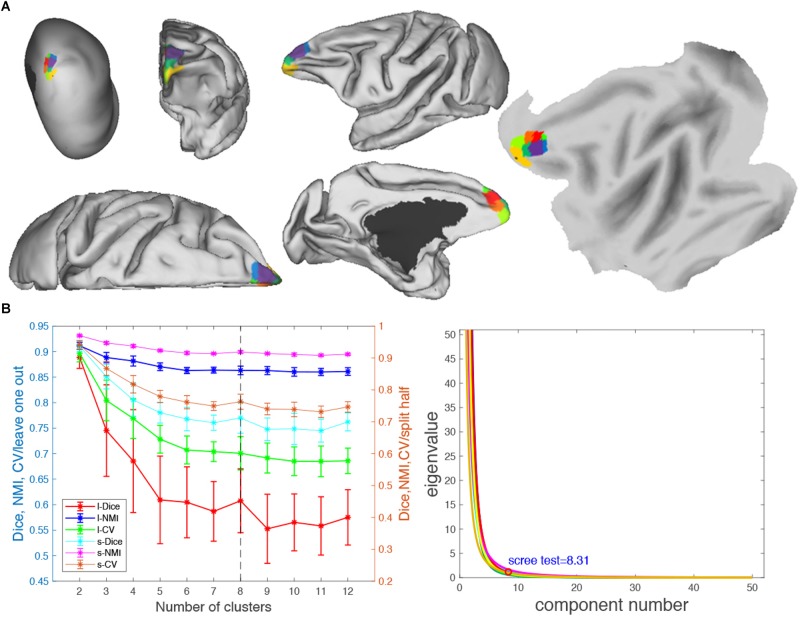
Parcellation results of left frontal pole (FP.L). **(A)** The MPMs of FP. L with 8 clusters in flat, fiducial (lateral, medial, anterior, superior) surface, very inflated (dorsal-lateral) view. **(B)** Validity indices of the parcellation results for FP.L. Thereinto, the results of Dice, NMI, and cross-validation (CV) using leave-one-out (l-Dice, l-NMI, and l-CV) and spilt-half (s-Dice, s-NMI, and s-V) resampling technique (left), and PCA (right) suggest that the optimal result of eight clusters seems the most reasonable. The error bars represent standard deviation.

**FIGURE 8:**
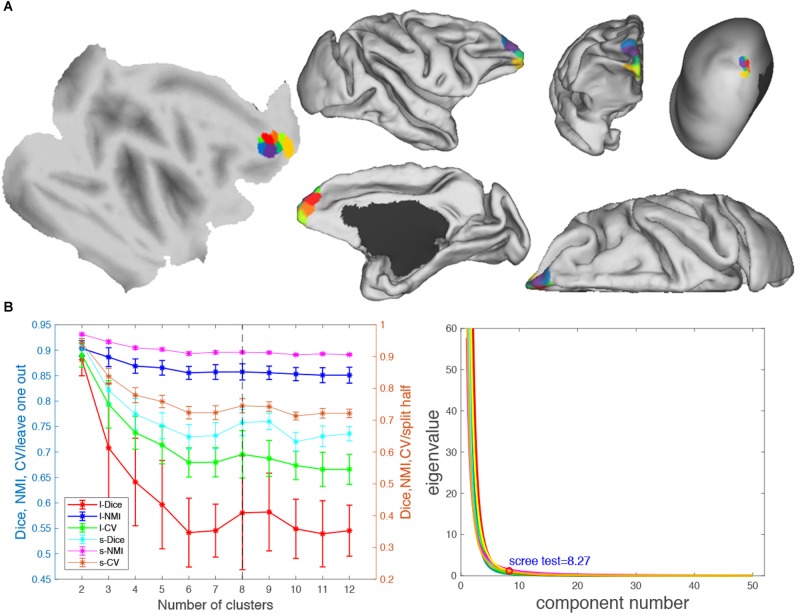
Parcellation results of right frontal pole (FP.R). **(A)** The maximum probability maps (MPMs) of FP. R with eight clusters in flat, fiducial (lateral, medial, anterior, and superior) surface, with very inflated (dorsal-lateral) view. **(B)** Validity indices of the parcellation results for FP.R. Thereinto, the results of Dice, NMI, and CV using leave-one-out (l-Dice, l-NMI, and l-CV) and spilt-half (s-Dice, s-NMI, and s-V) resampling technique (left) and principal component analysis (PCA) (right) suggest that the optimal result of right clusters seems the most reasonable. The error bars represent standard deviation.

**FIGURE 9:**
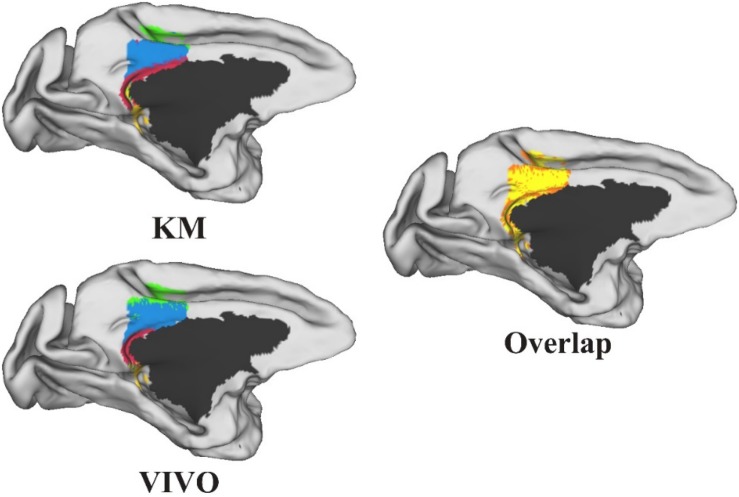
The parcellation results of the left posterior cingulate gyrus on the two datasets and the overlap between the two parcellation results. All the results were transformed and combined into F99 brain space. The yellow-shaded areas represent areas of overlap, and the orange-shaded areas represent the difference.

Performance Test: We tested MonkeyCBP on two batches of data in a local 12-node high-performance computing cluster, including six nodes that have 16 cores of Intel Xeon E5-2630@2.3GHz and 128GB memory and another six nodes that have 12 cores of Intel Xeon E5-2630@2.3GHz and 128GB memory. One dataset consists of eight monkeys (*Macaca mulatta*, two male and six female, 4–23 years old, weighted at 2.9–4.2 kg, and DWI with 0.6-mm isotropic voxels) obtained from Kunming Institute of Zoology, Chinese Academy of Sciences. All animals of Kunming were conducted according to policies set forth by the National Institutes of Health Guide for the Care and Use of Laboratory Animals and conformed to the protocol of the animal care and use committee of the Institute of Automation, CAS. To test the toolbox, we used the CLI and GUI version of MonkeyCBP to parcellate the right PCG. The parcellation results, including optimal number of subdivisions and stability indices, are shown in Figure 6 Then a high degree of consistency among the indicators can be observed. In addition, combined with other different indices, we parcellated the bilateral FP, and the simplified results are shown in Figures 7, 8. Wang et al. (2018) parcellated the cingulate cortex into eight anatomical areas and the frontal cortex into 14 anatomical areas. Further, MonkeyCBP found more subregions in different cortical areas, even small ones (e.g., the FP cortex). The parcellation results showed that the right PCG could be subdivided into four subregions and that the FP of each hemisphere could be subdivided into eight subregions, which is a finer parcellation result. It is worth mentioning that we have described the parcellation details of the FP in another paper that is under review. The time consumed of the entire process was approximately 5 and 3 h. Another dataset consists of five monkeys (*M. mulatta*) *in vivo* obtained from an open resource (Milham et al., 2018). To validate the stability of the parcellation scheme, we parcellated the left PCG on the basis of the two datasets. We calculated the number of all non-zero voxels in each postprocessed MPM, the number of overlapping voxels for the postprocessed MPM, and the number of overlapping voxels of each subregion. The overall proportion of overlapping voxels is 78.24%. Additionally, on each dataset, we calculated the proportion of overlapping voxels of each subregion; see Table 2 for more details. The parcellation results and the overlapping areas were transformed and combined into F99 brain space (Van Essen, 2002) using the Caret software (Van Essen et al., 2001) for visual inspection (Figure 9). Topological similarities in the parcellations between the two datasets can be observed.

**TABLE 2:** The overlapping ratios of the parcellations of the two datasets.

**Clusters**	**KM**			**VIVO**			**KM ∩ VIVO**		
	**Voxels**	**Overlap**	**Ratio (%)**	**Voxels**	**Overlap**	**Ratio (%)**	**Voxels**	**Overlap**	**Ratio (%)**
Cluster 1	987	811	82.17	1,130	811	71.77	–	–	–
Cluster 2	1,319	868	65.81	1,280	868	67.81	–	–	–
Cluster 3	785	289	36.82	373	289	77.48	–	–	–
Cluster 4	408	261	63.97	324	261	80.56	–	–	–
Total	3,499	2,229	–	3,107	2,229	–	2,849	2,229	78.24

*KM represents the macaque dataset from Kunming Institute of Zoology, Chinese Academy of Sciences. VIVO represents the open dataset of *in vivo* scanning. We noted that the overlapping ratio of cluster 3 on KM is 36.82% and identified significant difference in the position of the anterior posterior cingulate, adjacent to the corpus callosum. Considering the maximum possible overlap ratio would be 47.51% (373/785), the overlap ratio of 36.82% in cluster 3 was not as low as it looked like. In addition, the distribution pattern of parcellation results was consistent, and the total overlap is 78.24%, which suggested a good stability of the MonkeyCBP.*

To our best knowledge, this is the first toolbox dedicated for rhesus monkey brain parcellation based on connectivity profiles. On the basis of diffusion MRI data of macaque monkey *in vivo*, Wang et al. (2018) subdivided the monkey cortex into 80 subregions in each brain hemisphere using CBP. The registration was implemented using the FLIRT tool, and the cluster numbers were determined by continuity index. Wang et al. parcellated the frontal cortex into 14 subregions, the somatosensory cortex into 9 subregions, the parietal cortex into 13 subregions, the temporal cortex into 16 subregions, the occipital cortex into 16 subregions, and the limbic system into 12 subregions. In comparison, the two regions we have parcellated using MonkeyCBP were subdivided into finer regions, that is, the PCG and bilateral FP; we provide a segmentation result with more subdivisions. Although Wang et al. (2018) constructed the macaque cortex atlas using CBP, the pipeline was not published, and the automation performance of the process was not enough.

This toolbox inherited the advantages of ATPP, compared with other existing parcellation tools, including massive parallel computing within and across machines for high-throughput processing of high-resolution multimodal data, modularized software structure for easy extension and rapid development, and detailed intermediate results and abundant log information generated for quality control and reproducibility. MonkeyCBP follows open science protocol and is publicly accessible in Neuroimaging Informatics Tools and Resources Clearinghouse^3^ (NITRC)^4^; the Digital Object Identifiers (DOIs)^4^ (10.25790/bml0cm.56) are associated with MonkeyCBP. Its source codes are hosted in Github,^5,6^ under the GNU generic purpose license version 3^7^ (GPLv3), and are available for download and fork. Besides, to promote Resource Identification Initiative (Bandrowski et al., 2016), which aims to promote research resource identification, discovery, and reuse, Research Resource Identifier (RRID) was curated (MonkeyCBP, RRID:SCR_017640). by SciCrunch Resource Registry^8^ to avoid ambiguities on the tool name in addition to its version (Nichols et al., 2017).

Apart from the features mentioned above, MonkeyCBP includes a robust skull-stripping module for rhesus monkey. The deformable registration is performed using ANTs, instead of SPM, resulting in better registration. A PCA-based parcellation verification index is also included to exploit information from individual image space for accurate determination of number of parcels. The highly automated process and high-throughput performance supported by GPU option make the toolbox ready to be used by a wider research community.

## Data Availability Statement

The datasets presented in this article are not readily available because the data is still being used for scientific research. Requests to access the datasets should be directed to jiangtz@nlpr.ia.ac.cn.

## Author Contributions

All authors were responsible for design and prototyping of the pipeline, and reviewed and approved the final version of the manuscript. BH, ZY, and BG were responsible for the implementation of the pipeline. BH did the test experiment of the pipeline. ZY and BH drafted the manuscript.

## Conflict of Interest

The authors declare that the research was conducted in the absence of any commercial or financial relationships that could be construed as a potential conflict of interest.

## Footnotes

1 http://www.picsl.upenn.edu/ANTS/

2 https://mipav.cit.nih.gov/

3 https://www.nitrc.org

4 https://www.nitrc.org/projects/monkey-cbp

5 https://www.github.com

6 https://github.com/bheAI/MonkeyCBP

7 https://www.gnu.org/licenses/gpl-3.0.html

8 https://www.scicrunch.org/resources
